# Design and Synthesis of Pyrazole-3-one Derivatives as Hypoglycaemic Agents

**DOI:** 10.1155/2015/670181

**Published:** 2015-02-04

**Authors:** Prasanna A. Datar, Sonali R. Jadhav

**Affiliations:** Department of Pharmaceutical Chemistry, Sinhgad Institute of Pharmacy, Pune, Maharashtra 411041, India

## Abstract

Pyrazole-3-one compounds were designed on the basis of docking studies of previously reported antidiabetic pyrazole compounds. The amino acid residues found during docking studies were used as guidelines for the modification of aromatic substitutions on pyrazole-3-one structure. Depending on the docking score, the designed compounds were selectively prioritized for synthesis. The synthesized compounds were subjected to *in vivo* hypoglycemic activity using alloxan induced diabetic rats and metformin as a standard. Compound 4 having sulphonamide derivative was found to be the most potent compound among the series.

## 1. Introduction

Diabetes mellitus (DM) is a metabolic disorder resulting from a defect in insulin secretion, insulin action, or both. Insulin deficiency in turn leads to chronic hyperglycaemia with disturbances of carbohydrate, fat, and protein metabolism [1]. Diabetes is the most common endocrine disorder, that is, multifaceted condition that includes *β*-cell dysfunction, neurotransmitter dysfunction, decreased insulin secretion, decreased incretin effect, increased hepatic glucose production, increased glucagon secretion, decreased glucose uptake, peripheral insulin resistance, and elevated hepatic gluconeogenesis together with hypertension, dyslipidemia, obesity, and cardiovascular disease [2]. As the disease progresses, tissue or vascular damage ensues leading to severe diabetic complications such as retinopathy, neuropathy, and nephropathy [3]. The prevalence of diabetes is rising all over the world due to population growth, aging, urbanisation, and an increase of obesity and physical inactivity. The International Diabetes Federation (IDF) estimates the total number of people in India with diabetes to be around 50.8 million in 2010 rising to 87.0 million by 2030 [4]. Type 2 diabetes mellitus (T2DM) presents a major challenge to healthcare system around the world. The current oral treatment options for T2DM include metformin, sulfonylurea or thiazolidinedione derivatives, glycosidase inhibitors, and the recently dipeptidyl peptidase IV inhibitors which have been introduced. Antioxidants are used as supportive therapy in the treatment of DM and hypoglycemic plants have been shown to regulate the oxidative complications of DM [5].

Pyrazole derivatives are the subject of many research studies due to their widespread potential biological activities such as analgesic [6, 7], antidiabetic [8], anticonvulsant [9], antimicrobial [10], anti-inflammatory [11], antiviral [12], and anticancer activities [13]. 1,5-Diarylpyrazole derivatives have been reported as nonnucleoside HIV-1 reverse transcriptase inhibitory activity [14]. Kees et al. [15] have described (4-substituted benzyl) (trifluoromethyl)pyrazoles and pyrazolones which represent a new class of antidiabetic compounds. Cottineau et al. [16] have reported substituted pyrazole-4-carboxylic acid and reported the pharmacophore 3-methoxy-1*H*-pyrazole-4-carboxylic acid ethyl ester as a hypoglycemic agent. Das et al. [17] have synthesized substituted pyrazole-3-one derivatives as potential hypoglycaemic agent.

## 2. Experimental Methods

### 2.1. Molecular Docking Study of Pyrazole Derivatives

Molecular docking studies were performed by using Glide v5.6 (Schrodinger, LLC) [18]. The X-ray crystal of peroxisome proliferator-activated receptor gamma (PPARgamma) in complex with rosiglitazone (PDB entry code 1FM6) [19] was obtained from the RCSB Protein Data Bank (PDB) and utilized in order to get the detailed insights of ligand-protein structure in this study. Out of four chains in the reported protein structure 1FM6, only single monomer chain with rosiglitazone was selected for further work. Hydrogen atoms were added to the protein structure and were energetically optimized. Docking study was performed in single monomer by replacing rosiglitazone from reported crystal structure 1FM6. In case of docking poses showing high score, some manual adjustments were done to remove large steric hindrances and final complex structures were subjected to energy minimization using OPLS force field in Schrodinger software. During the energy minimization procedure, whole protein structure was fixed except the region within 3.5 Å radius from each ligand which was relaxed.

Several pyrazole compounds have been reported for their antidiabetic effect by acting as agonist at PPARgamma [18]. Taking the guidelines of reported ligand-receptor interaction obtained from docking studies of pyrazole compounds with PPARgamma protein, the study was extended on docking study of pyrazole compounds that were reported as antidiabetic without PPARgamma agonism [16, 17] as shown in Tables 1 and 2.

Rosiglitazone was removed from the complex and the reported pyrazole compounds were placed in the same cavity and were further docked in PPARgamma protein and their binding interaction was validated with previously reported binding residues [18]. These binding residues were used as reference guide. Docking was performed selectively on reported pyrazole analogues (Tables 1 and 2) for which antidiabetic activity is known to be potent and comparable to standard. This study helped in understanding possible orientations and conformation of pyrazole compounds and their resulting position and binding interaction in the pocket. Similar to the thiazolidinedione group of rosiglitazone, the carboxyl group of reference compound was found to show hydrogen bonds with Tyr473 as well as Ser289 and His449. Further docking of newly designed virtual pyrazole compounds (Table 3) was performed. Docking positions with a high score were corrected manually to avoid small steric bumps, minimized energetically using OPLS force field.

### 2.2. G-Score

The docking studies were performed using standard precision mode of Glide. The results of the docking studies were generated in the form of G-score. The more negative value of G-score means the compound is more potent and indicates good binding potential. Besides the G-score, other parameters like energy and E-model were also taken into consideration for the evaluation of the docking results. The values of energy and E-model were found to be significantly closer to the values of the standard rosiglitazone.

### 2.3. H-Bond Interaction

As H-bond is an influential parameter, H-bonding governs binding affinity of the docked compounds. The number of H-bond interactions in the standard compound rosiglitazone was compared with those of the designed virtual pyrazole compounds. In case of docking of the standard compound rosiglitazone with 1FM6, only one H-bond interaction was found.

New ligands were designed virtually to improve the binding interaction in terms of improving the hydrogen binding capability of ligand along with variation in bulkiness at ortho-, meta-, and parapositions of aromatic ring. On the basis of docking study, amino acids such as Ser342, Tyr473, and Ser289 were found to preserve binding interaction with the designed virtual molecules. The choice of best-docked structure for each ligand is made using a model energy score (*E-model*) that combines the energy grid score, the binding affinity predicted by GlideScore, potential energy, lipophilicity, hydrogen bond, and van der Walls energy. On the basis of these parameters, six compounds were selected for synthesis. Table 3 shows designed virtual molecules 1, 3–6, while Compound 2 is reported in PubChem Compound Database (CID 20269015).

### 2.4. Chemistry

In light of the above, a new series of aryl substituted pyrazole-3-one derivatives were synthesized and evaluated for their possible hypoglycaemic activity. Substituted phenylhydrazines were prepared from anilines by diazotization. Pyrazole-3-one-4-carboxylate derivatives were then generated by reacting diethylethoxymethylene malonate (DEEM) with substituted phenyl hydrazine through base catalyzed cyclization reaction. The compounds were synthesized by Michael addition reaction, which is a nucleophilic addition of enolate anions to the carbon-carbon double bond of *α*,*β*-unsaturated carboxylic acid derivatives. The synthesized compounds were tested for hypoglycaemic activity.

### 2.5. Experimental

All reagents and anhydrous solvents were generally used as they are received from the commercial supplier. Reactions were routinely performed in oven-dried glassware. Melting points were determined with Veego VMP-D digital melting point apparatus and are uncorrected. FTIR spectra were recorded on a JASCO FTIR 4100 series spectrophotometer and are reported in cm^−1^. ^1^H NMR spectra were recorded on Varian 300 MHz in DMSO unless otherwise specified and chemical shifts are reported relative to tetramethylsilane as an internal standard.

### 2.6. Scheme of Synthesis

See Figure 1.

### 2.7. General Procedure for Synthesis of Compounds 1, 3–6

*Preparation of Substituted Phenyl Hydrazine from Substituted Aniline.* 5 g (0.037 mol) of substituted amino compound was dissolved in a mixture of 10.5 mL of concentrated HCl and an equal volume of water, cooled rapidly to 0°C in order to obtain the hydrochloride of the base in a fine state of division. Gradual addition of a solution of 2.6 g (0.037 mol) of sodium nitrite in 6 mL of water was performed for diazotization. Stirring was continued for a few minutes, and the solution was filtered and added by using a separatory funnel to an ice-cold solution made of 41 g (0.156 mol) of sodium sulphite (96% Na_2_SO_3_·7H_2_O) in 100 mL of water containing 4 g of NaOH. The solution was allowed to stand for 5 minutes, acidify with 35 mL of concentrated HCl, and heat on a water bath at 25°C for 3 minutes, when yellow needles commence separating. This solution was kept overnight, filtered off the crystals, heated with 10 mL of concentrated HCl on a water bath for 7 minutes, and allowed to cool. The precipitate was filtered off and dissolved in water and the solution was treated with a concentrated solution of sodium acetate. The free base separated out in an almost pure state [20].

### 2.8. Synthesis of Aryl Substituted-1H-pyrazole-3-one-4-carboxylate

*Procedure.* Substituted phenyl hydrazine (0.02 mol) was dissolved in minimum amount of cold water and then ethanolic KOH was added. The solution was then refluxed for 40 min at 70°C in the presence of diethylethoxymethylene malonate (DEEM) (0.02 mol). The precipitate obtained was filtered, washed with water, and dried. The product obtained was recrystallized from ethanol and was dried for 24 hr at room temperature and kept in a vacuum desiccator [17].

### 2.9. Antidiabetic Activity

*In Vivo Studies [21].* Fasting blood glucose was determined after depriving food for 16 hr with free access for drinking water. Initially doses of alloxan 40, 50, 65, 90, and 120 mg/kg were given to six rats each and induction of diabetes was observed. Hyperglycemia was induced by a single i.p. injection of 90 mg/kg of alloxan monohydrate (Explicit Chemicals, India) in sterile saline. After 5 days of alloxan injection, the hyperglycemic rats (glucose level > 200 mg/dL) were separated and divided into different groups comprising six rats each for the antidiabetic study. The synthesized compounds were administered on 5th day. The dose was decided on the basis of the literature. Rats in control group were given standard diet. Treatment of synthesized compound was started from the same day using 1% CMC with the dose of 40 mg/kg (p.o.). The dose was decided as per the reported study of homologous series of pyrazole-3-one compounds [17]. Another group of six rats were given metformin as standard drug by administering dose of 120 mg/kg. During this period, animals in all groups had free access to standard diet and water. The blood was withdrawn by retroorbital plexus method [22]. On the same day, the blood glucose levels were estimated by using glucometer (Johnson & Johnson Pvt. Ltd.) with one-touch simple select strips. The blood glucose levels were estimated during 1 hr, 3 hr, and 6 hr. Out of 48 rats, 45 rats survived. The dead rats were disposed of as per standard protocol of CPCSEA rules.

## 3. Results

### 3.1. Molecular Docking Study of Pyrazole Compounds

Initially docking of rosiglitazone was validated with docking studies. Binding behavior of reported antidiabetic pyrazole compounds was studied due to their structural difference from thiazolidinedione. Number of hydrogen binding ability of pyrazole derivatives was different from thiazolidinediones and hence comparison between two different scaffolds was not possible in terms of hydrogen-bonding interaction. Only common residues can be identified.

Docking results of reported pyrazole molecules and their binding interactions with residues within 3 Å radius were found out to understand the specific type of binding interactions of molecular features of pyrazole compounds [16, 17]. G-score value obtained after docking study was compared with antidiabetic activity of each of reported reference pyrazole compounds. G-score of potent pyrazoles was found to be correlated. Thereby crucial residues of binding interaction among the potent reported pyrazole compounds were identified. The presence or absence of these crucial residues was observed in docking study of novel pyrazole-3-one compounds 1, 3–6.

**Figure 2:**
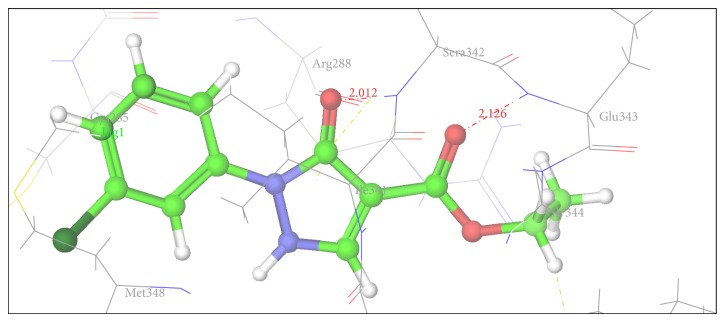
Docking pose of Compound 1 having interaction with Ser342 and Glu343.

**Figure 3:**
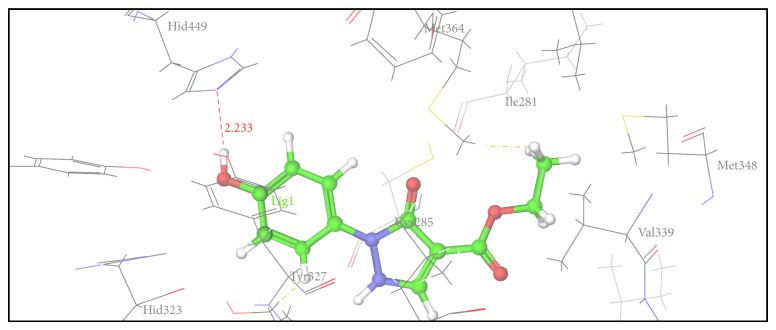
Docking pose of Compound 3 having interaction with His449.

**Figure 4:**
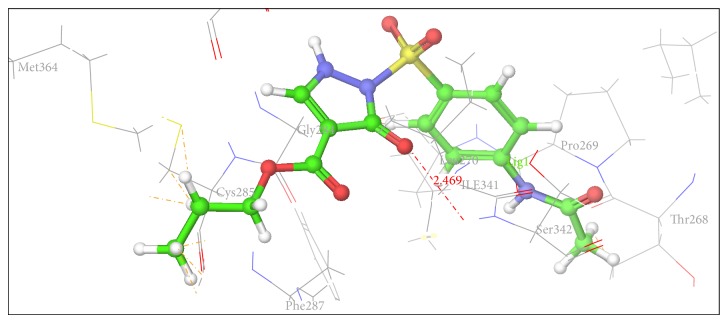
Docking pose of Compound 4 having interaction with Ser342.

**Figure 5:**
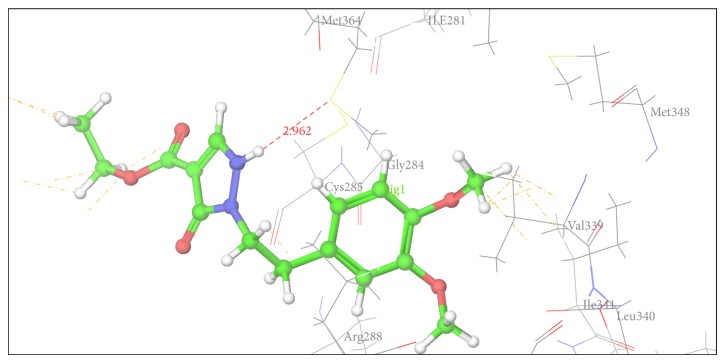
Docking pose of Compound 5 having interaction with Met364.

**Figure 6:**
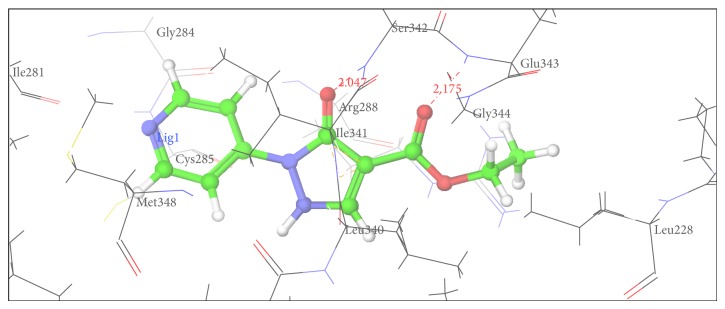
Docking pose of Compound 6 having interaction with Ser342 and Glu343.

**Figure 7:**
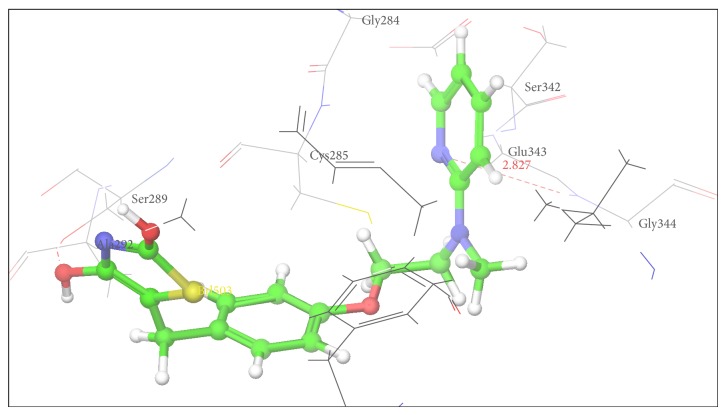
Docking pose of rosiglitazone having interaction with Ser342 and Ser289.

Figures 2, 3, 4, 5, 6, and 7 show the docked compounds (1, 3, 4, 5, and 6, rosiglitazone) in the active site of 1FM6, respectively. The ligands are shown colored in atom type with ball and stick representation.

Table 4 shows results of docking of reported pyrazole-3-one molecules showing hydrogen-bonding interaction with Ser289, Ser342, and Glu343.  Table 5 shows results of docking of reported carboxylate anion of pyrazole-4-carboxylic acid derivatives that act as an anchor to bind to the amino acid residues such as Ser289, Ser342, and Tyr473. Thus, Tables 4 and 5 show common residues such as Ser342 and Ser289. From these results, it was observed that both the reported pyrazole compounds have common binding interaction with Ser289 as that of rosiglitazone.  Table 6 shows amino acid interactions within 3 Å distance for each designed compound (1, 3–6). This region of binding is similar to binding region of rosiglitazone except for Compound 2, which drifts away from this region.

By considering the docking parameters such as docking score, electrostatic coulomb, and van der Waals energy parameters potency of the compounds was estimated.

In comparison to position of rosiglitazone, in the docking model of the literature based Compound 2 (CID 20269015), the pyrazole ring slightly shifts away due to binding interaction of –OCH_3_ group at His 449. Similarly in case of designed Compound 3 presence of phenolic OH group at R position fell to reach the important binding interaction. Compound 5 shows large number of bad van der Waals contacts resembling the strained binding interaction with His449 due to two methoxy substituents on aromatic ring at R. In Compound 6, presence of pyridine ring at R shifts away the molecule leading to nonbonding attraction of electronegative N atom. Compound 1 showed close anchoring of carboxylate ion with the Glu343 and Ser342 residues. This anchoring is very similar to the binding interaction observed in pyrazole-4-carboxylic acid reference structures as well as propionic acid derivatives reported in crystal structure. In Compound 4, introduction of 4-acetamidobenzenesulphonamide was expected to form increase in one or two hydrogen bonds. As shown in Figure 3, 4-acetamidobenzenesulphonamide group exhibited hydrogen bond with Ser342 via the carbonyl oxygen of pyrazole-3-one ring. The docking study of Compound 4 showed hydrogen bonding with carbonyl group of pyrazole-3-one due to particular orientation and flexibility of 4-acetamidobenzenesulphonamide group which might have made carbonyl group of pyrazole-3-one accessible for crucial binding. This interaction is absent in all other compounds. These docking results suggest that the pyrazole substituted 4-acetamidobenzenesulphonamides are a new class of selective PPARgamma agonists.  Table 7 shows hydrogen bonding of the designed molecules with amino acid residue.

### 3.2. Characterization of Synthesized Compounds

1. * Compound 1.* Yield: 32.45%; m.p. 260-261°C; Rf value (0.53), IR(KBr): 3288.04 (NH str), 1706.69 (–CO str of ester), 1679.68 (–C=O), 1590.99 (Ar-C-C-str), 752.10 (Cl); ^1^H NMR (DMSO): *δ* 2.5 (s, 3H, –CH_3_), 4.0 (q, 2H, –OCH_2_), 7.6–8.0 (m, 4H, Ar-H), 8.6 (s, 1H, –NH).
2. * Compound 3.* Yield: 17.17%; m.p. 103-104°C; Rf value (0.87), IR(KBr): 3517.52 (–OH), 3460.63 (NH str.), 1772.25 (–CO str of ester), 1606.45 (–C=O); ^1^H NMR (DMSO): *δ* 2.46 (s, 3H, –CH_3_), 3.9 (q, 2H, –OCH_2_), 5.2 (s, 1H, –OH), 7.72 (m, 4H, Ar-H), 8.5 (s, 1H, –NH).
3. * Compound 4.* Yield: 29.46%; m.p. 167-168°C, Rf value (0.84), IR(KBr): 3370 (NH str.), 1658.48 (–C=O), 1594.84 (Ar-C-C-Str.), 1260.25 (–NH amide), 1037.52 (–S–); ^1^H NMR (DMSO): *δ* 2.45 (s, 6H, –CH_3_), 4.2 (q, 2H, –OCH_2_), 6.8 (m, 2H, Ar-H), 7.2 (m, 2H, Ar-H), 8.5 (s, 1H, –NH), 10.6 (s, 1H, –CONH).
4. * Compound 5.* Yield: 37.87%; Semisolid, Rf value (0.59), IR(KBr): 3328.53 (NH str.), 1742.37 (C=O ester), 1702.84 (–C=O), 1529.27 (Ar-C-C-Str.), 1213.97 (–CO ether); ^1^H NMR (DMSO): *δ* 2.6 (t, 3H, –CH_3_), 3.5 (4H, m, –CH_2_), 3.9 (s, 6H, –OCH_3_), 4.20 (q, 2H, –OCH_2_), 6.65–7.2 (m, 4H, Ar-H), 9.25 (s, 1H, –NH).
5. * Compound 6.* Yield: 16.77%, Semisolid, Rf value (0.49), IR(KBr): 3361 (NH str.), 1746 (C=O ester), 1711 (–C=O), 1304 (CN); ^1^H NMR (DMSO): *δ* 2.45 (t, 3H, –CH_3_), 4.19 (q, 2H, –OCH_2_), 8.4 (m, 4H, pyridine), 9.2 (s, 1H, –NH).

### 3.3. Antidiabetic Activity:* In Vivo* Studies

Table 8 shows blood glucose level which is expressed in mg/dL where Compounds 1 and 4 showed more potency.

**Figure 8:**
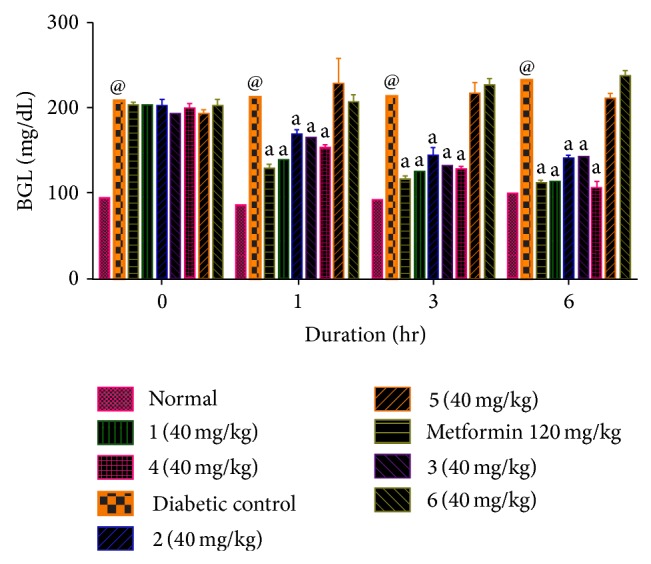
Effect of metformin 120 mg/kg p.o. and 1, 2, 3, 4, 5, and 6 (40 mg/kg, p.o.) on blood glucose level. Values in parentheses indicate the dose in mg/kg. Data are expressed as mean ± SEM and analyzed by Bonferroni post hoc test, using two-way ANOVA, *n* = 6 rats per group; ^a^ *P* < 0.001, as compared to diabetic control, and ^@^ *P* < 0.001 as compared to normal.

In Figure 8, the graph that expresses blood glucose level for intervals of 1, 3, and 6 hr and data was analyzed by Bonferroni post hoc test, using two-way ANOVA method [21].

## 4. Discussion

The results of the docking studies are presented in the form of G-score. The G-scores are presented as negative values, indicating that higher negative values resemble higher binding interaction. The docking studies were performed for the designed pyrazoles (as shown in Table 7) with PPARgamma enzyme, and the results were compared with rosiglitazone and reported pyrazole compounds present within the receptor. The docked complexes of the designed compounds along with the ligand receptor poses showed ligand occupying similar binding pocket as that of rosiglitazone although differed in their binding to different amino acid residues. The designed compounds were found to display good binding affinity to the receptor in terms of hydrogen binding and G-score values. Red dotted lines in Figures 2 to 7 indicate H-bond interactions with receptor.* In vivo* studies of alloxan induced diabetes rat model show that Compounds 1 and 4 have more potency while Compounds 2 (CID 20269015) and 3 have moderate potency compared to standard. Compounds 5 and 6 were least active. The results were calculated by measuring the mean ± SEM “*P*” value and analyzed by Bonferroni post hoc test, using two-way ANOVA (Graph pad, prism software version 4.03, USA).

On the basis of docking results, Compound 1 was having more potency than Compound 4, but according to* in vivo* study, we found that Compound 4 has more potency over Compound 1. This small difference in potency of Compounds 1 and 4 was not expressed in terms of G-score. As per docking results, Compounds 2, 3, 5, and 6 showed poor binding affinity which has been reflected in the* in vivo* study. Thus, results were matching with actual experimental studies.

## 5. Conclusion

The pyrazole compounds were designed to have antidiabetic activity using docking studies. Docking study has revealed crucial hydrogen binding of carbonyl oxygen of pyrazole-3-one compounds. Docking studies have also helped in reducing synthetic work thereby saving time and chemical expenditure. Experimental* in vivo* study results matched with docking study. On the basis of docking studies using PPARgamma protein, we cannot claim the above compounds to be active for PPARgamma but they are antidiabetic based on* in vivo* experimental studies.

## Supplementary Material

Substituted pyrazole 3-one compounds were evaluated by FTIR and proton NMR stuides. FTIR spectra were recorded on a Jasco FTIR 4100 series spectrophotometer and are reported in cm-1. 1H NMR spectra were recorded on Varian 300 MHz in DMSO unless otherwise specified and chemical shifts are reported relative to tetramethylsilane as an internal standard. The series of compound have common feature such as ethyl ester group, carbonyl group, secondary and tertiary nitrogen and an aromatic center. Purity of the compounds was confirmed by absence of peak of NH2 group in FTIR so as the final compounds were devoid of impurities of starting material of substituted aniline.

## Figures and Tables

**Scheme 1:**
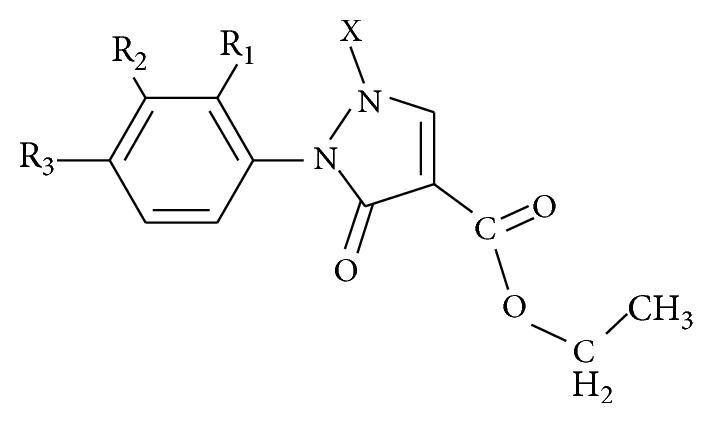


**Scheme 2:**
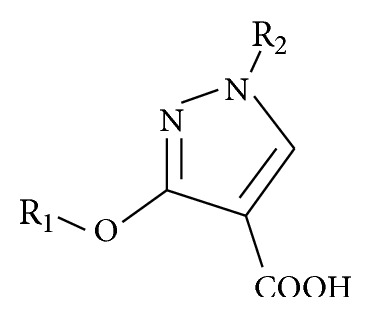


**Scheme 3:**
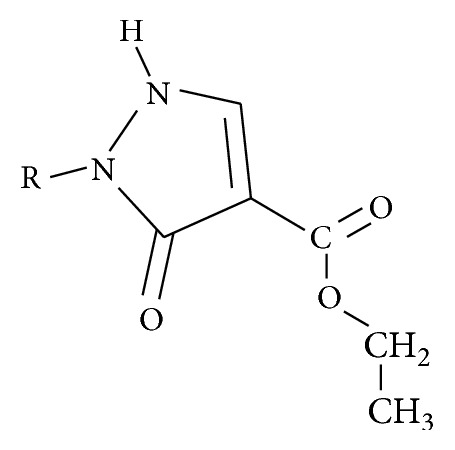


**Figure 1:**
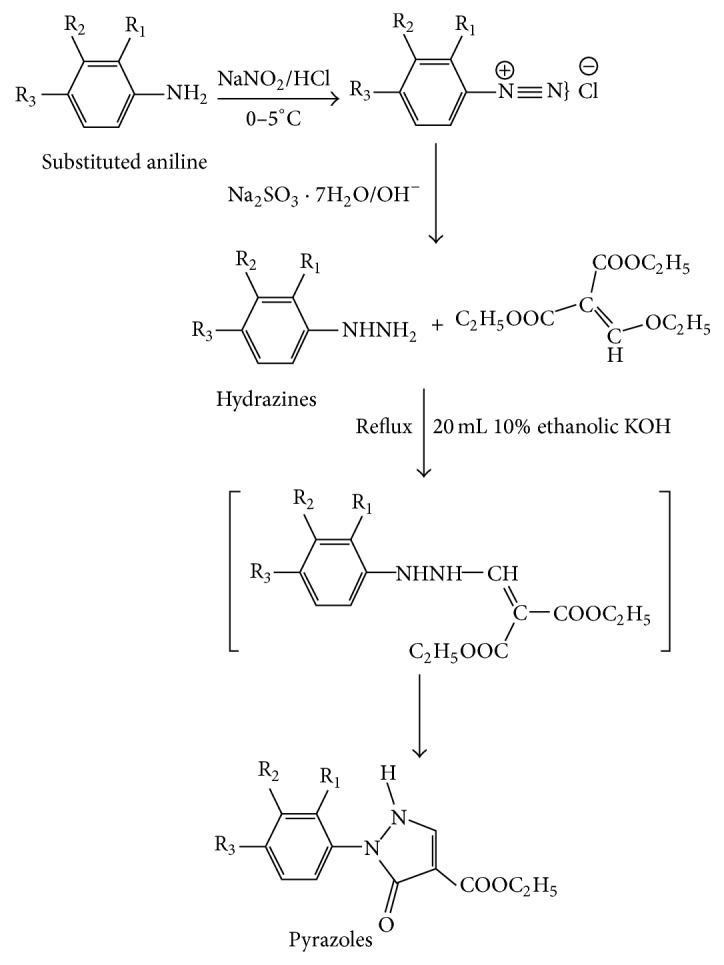
Scheme of synthesis of pyrazole-3-one derivatives.

**Table 1:** Reported antidiabetic pyrazole-3-one compounds used for docking studies.

Reference compound	R_1_	R_2_	R_3_	X

R1	H	H	H	H
R2	H	H	H	C_6_H_5_
R3	H	H	NO_2_	H

See Scheme 1.

**Table 2:** Reported antidiabetic pyrazole-4-carboxylic acid compounds used for docking studies.

Reference compound	R_1_	R_2_
R4	H	H
R5	CH_3_	H
R6	2-CN-[1,1′-biphenyl]-4′-(CH_2_)	H
R7	CH_3_	(CH_3_)_2_C=CH-CH_2_
R8	C_6_H_5_CH_2_	CH_3_CH_2_OOC-(CH_2_)_3_
R9	C_6_H_5_CH_2_	Tetrazole-CH_2_
R10	H	CH_3_

See Scheme 2.

**Table 3:** Newly designed pyrazole-3-one compounds.

Compounds	R

1	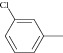
2	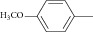
3	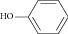
4	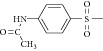
5	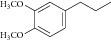
6	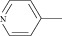

See Scheme 3.

**Table 4:** Results of docking studies for reported pyrazole-3-one molecules and their binding interactions with residues within 3 Å radius.

Reference compound	Number of hydrogen bonds	Hydrogen bond distance	Group of compounds	Group of amino acids	Residue number
R1	2	2.022	–C=O	–NH	Ser342
		2.094	–C=O	–NH	Glu343
R2	1	2.470	–C=O	–NH	Ser342
R3	2	1.982	–C=O	–NH	Ser342
		2.066	–C=O	–NH	Glu343

**Table 5:** Results of docking studies for reported pyrazole-4-carboxylic acid molecules and their binding interactions with residues within 3 Å radius.

Reference compound	Number of hydrogen bonds	Hydrogen bond distance	Functional group of compounds	Functional group of amino acids	Residue number
R4	1	2.280	–N	–OH	Ser289
R5	1	2.639	–C=O	–OH	Ser289
R6	1	2.659	–CN	–NH	Ser342
R7	1	2.138	–O–CH_3_	–OH	Tyr473
R8	1	2.824	–OH	–C=O	Leu340
R9	2	2.606	–OH	–O–C=O	Glu291
		2.067	–NH	–C=O	Gln271
R10	1	2.324	–C=O	–OH	Ser289

**Table 6:** Amino acid interactions within 3 Å distance from the compound.

Compound	Amino acid interaction						
1	Ser 342	Glu 343	Gly 344	Arg 288	Cys 285	Met 348	Ile 341
	∗	∗	—	—	—	—	—

3	His 449	Tyr 327	Cys 285	Ile 281	Val 339	Met 348	His 323
	∗	—	—	—	—	—	—

4	Pro 269	Ser 342	Thr 268	Gly 264	Cys 285	Phe 287	Leu 270
	—	∗	—	—	—	—	—

5	Met 288	Ile 281	Cys 285	Gly 284	Arg 288	Val 339	Leu 340
	∗	—	—	—	—	—	—

6	Ser 342	Glu 343	Gly 344	Leu 340	Cys 285	Gly 284	Met 348
	∗	∗	—	—	—	—	—

Rosiglitazone	Glu 343	Ser 342	Cys 285	Gly 284	Ser 289	Gly 344	Ala 292
	—	∗	—	—	∗	—	—

— means absence of interaction; ∗ means interaction.

**Table 7:** H-bonding interactions and G-score results of docking studies of designed molecules.

Compound	H-bond		Number of H-bonds	G-score
	Amino acid	Distance (Å)		
1	Ser342	2.019	2	−8.04
	Glu343	2.126		
3	His446	2.233	1	−7.23
4	Ser342	2.489	1	−7.29
5	Met364	2.962	1	−8.02
6	Ser342	2.047	2	−7.25
	Glu343	2.175		
Rosiglitazone	Ser342	2.827	1	−9.94

**Table 8:** Biological evaluation of compounds on Wistar rats.

Drug	Blood glucose level mg/dL (mean ± SE)			
	0 hr	1 hr	3 hr	6 hr
Normal	90.667 ± 1.585	90.667 ± 1.585	92.00 ± 1.438	91.500 ± 1.996
1	201.333 ± 4.379	137.333 ± 2.963	123.667 ± 3.461	112.833 ± 4.909
2 (CID 20269015)	201.833 ± 4.778	167.833 ± 3.468	151.00 ± 2.098	139.833 ± 2.358
3	198.333 ± 9.584	155.167 ± 3.321	136.833 ± 2.197	125.00 ± 4.139
4	198.667 ± 2.629	151.500 ± 1.648	125.833 ± 1.887	107.333 ± 3.658
5	190.00 ± 5.471	226.667 ± 28.273	215.167 ± 10.150	210.00 ± 3.873
6	200.00 ± 7.038	204.667 ± 7.292	225.333 ± 5.719	235.833 ± 4.527
Diabetic control	206.167 ± 5.747	210.667 ± 4.364	212.00 ± 4.494	229.667 ± 5.942
Metformin	201.167 ± 2.120	127.333 ± 3.621	114.833 ± 3.219	110.500 ± 1.607
