# Supplementary material for: Design and Synthesis of Pyrazole-3-one Derivatives as Hypoglycaemic Agents
Source: Int J Med Chem. 2015 Feb 4;2015:670181. doi: 10.1155/2015/670181 (PMC4334925; doi:10.1155/2015/670181)
Supplement: Supplementary file 1 — Substituted pyrazole 3-one compounds were evaluated by FTIR and proton NMR stuides. FTIR spectra were recorded on a Jasco FTIR 4100 series spectrophotometer and are reported in cm-1. 1H NMR spectra were recorded on Varian 300 MHz in DMSO unless otherwise specified and chemical shifts are reported relative to tetramethylsilane as an internal standard. The series of compound have common feature such as ethyl ester group, carbonyl group, secondary and tertiary nitrogen and an aromatic center. Purity of the compounds was confirmed by absence of peak of NH2 group in FTIR so as the final compounds were devoid of impurities of starting material of substituted aniline. [file 670181.f1.doc]

**Ms. Ref. No.: JTUSCI-D-14-00093R1**

**IR Spectra of compound 1:**


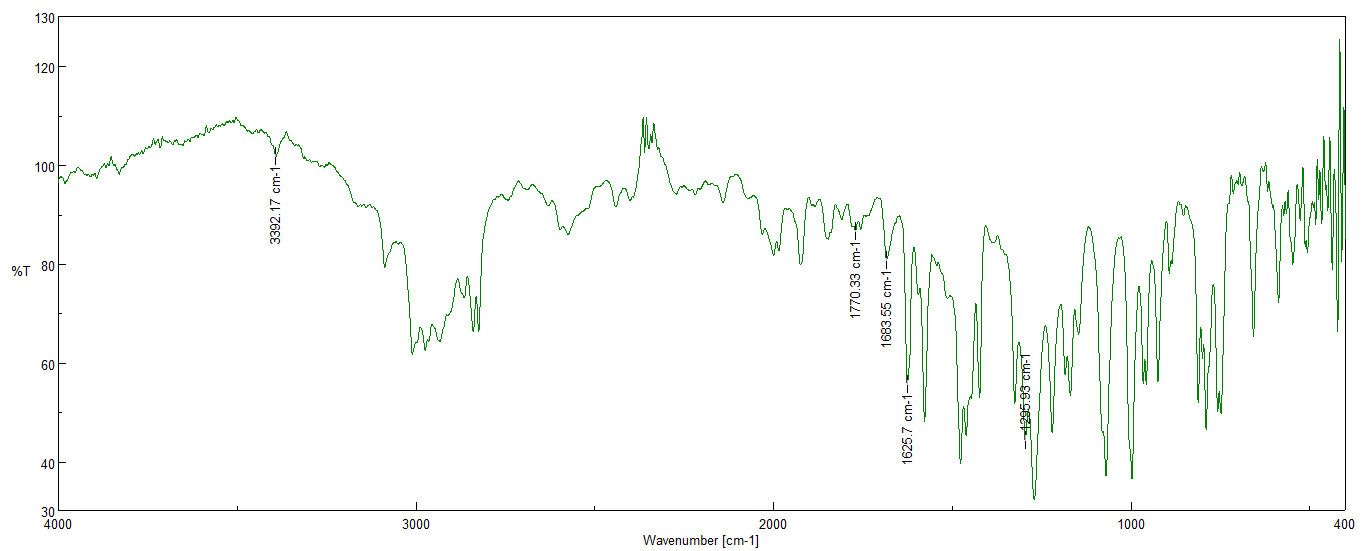


**NMR Spectra of compound 1:**


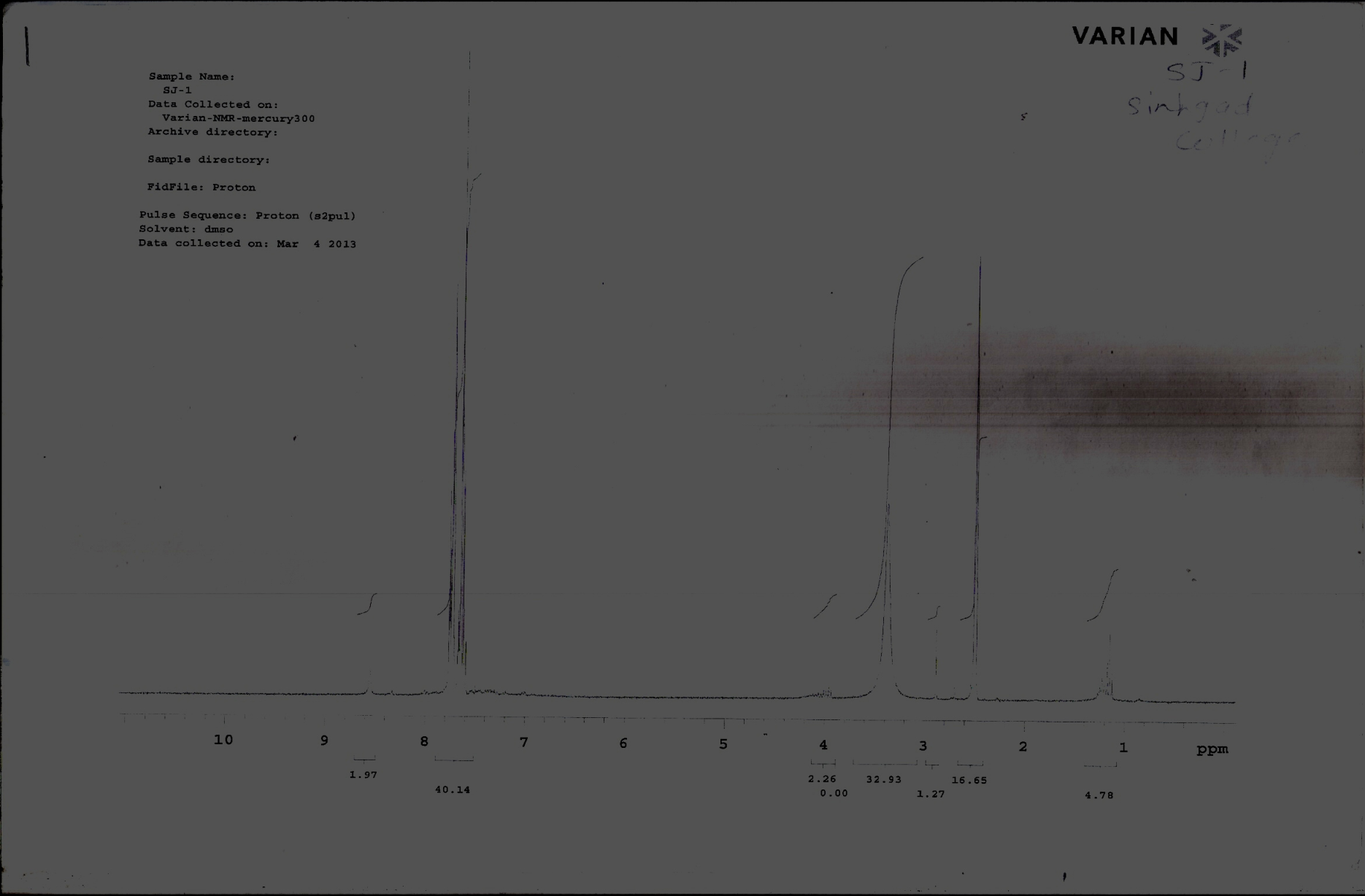


**IR Spectra of compound 2:**

**
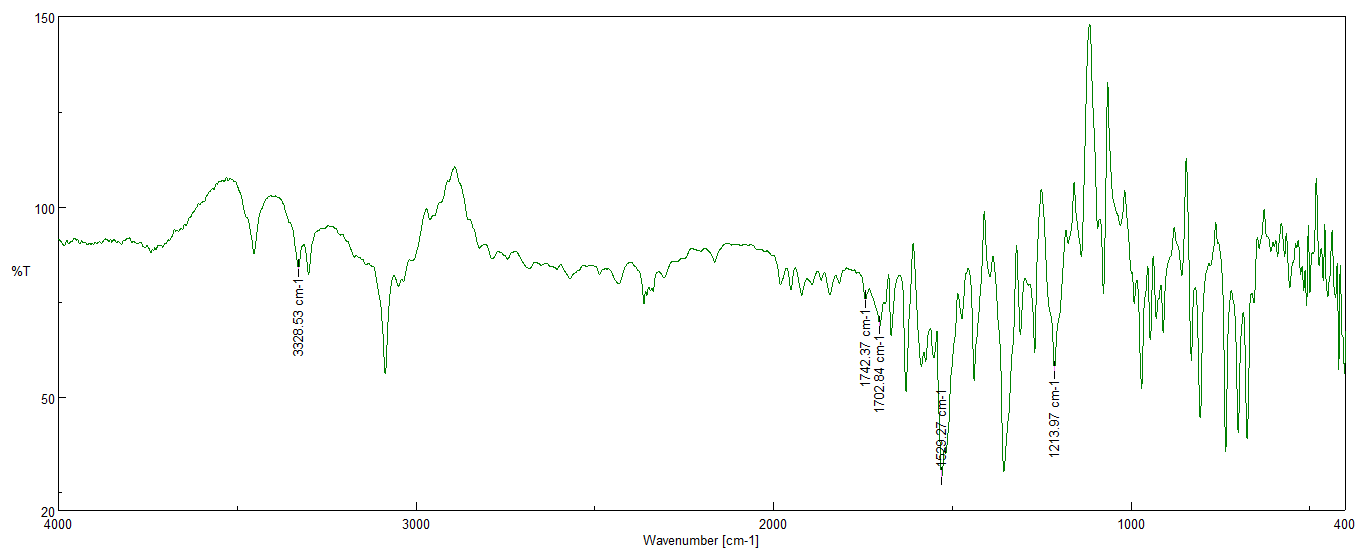
**

**NMR spectra of compound 2:**

**
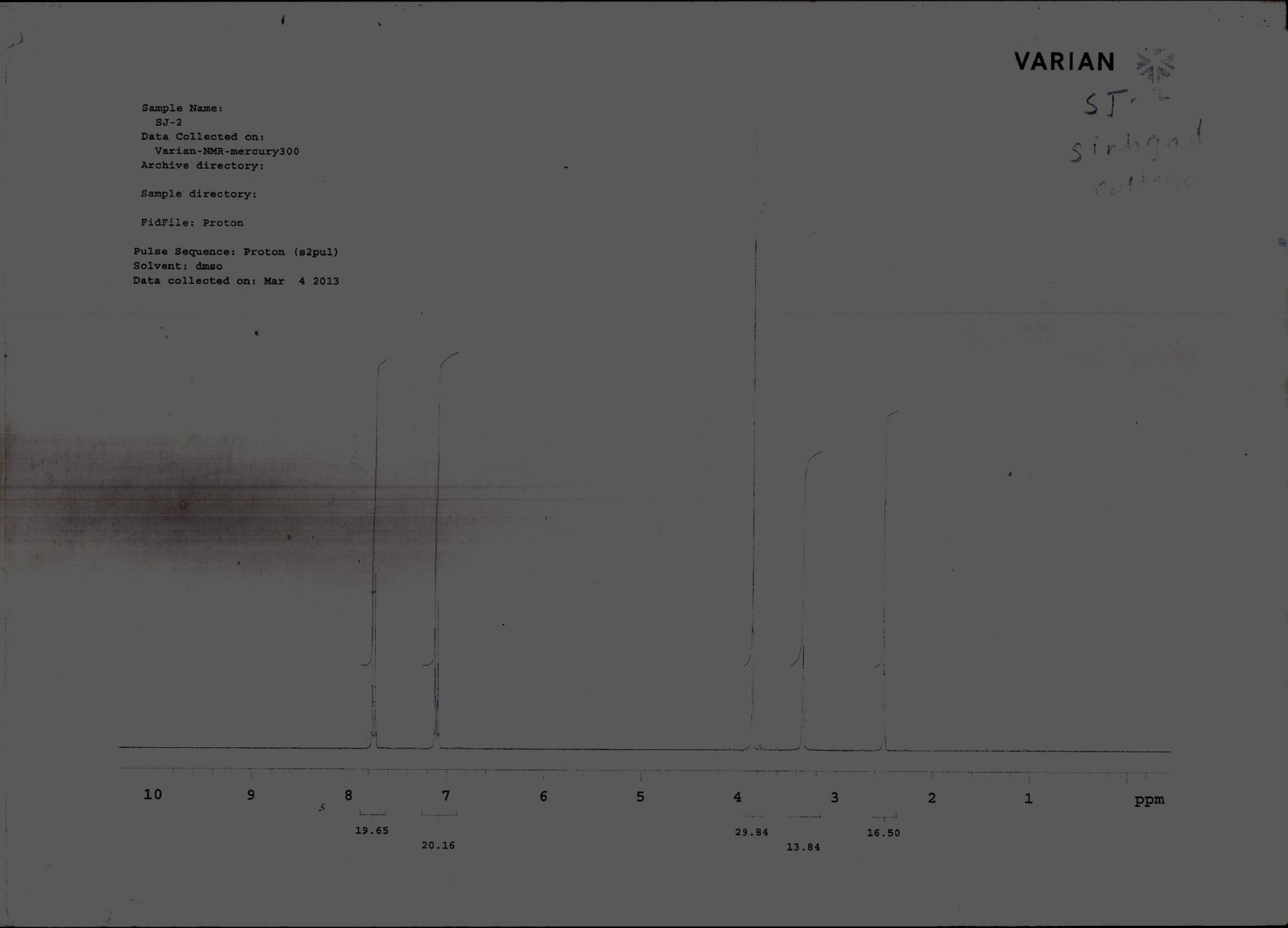
**

**IR Spectra of compound 3:**

**
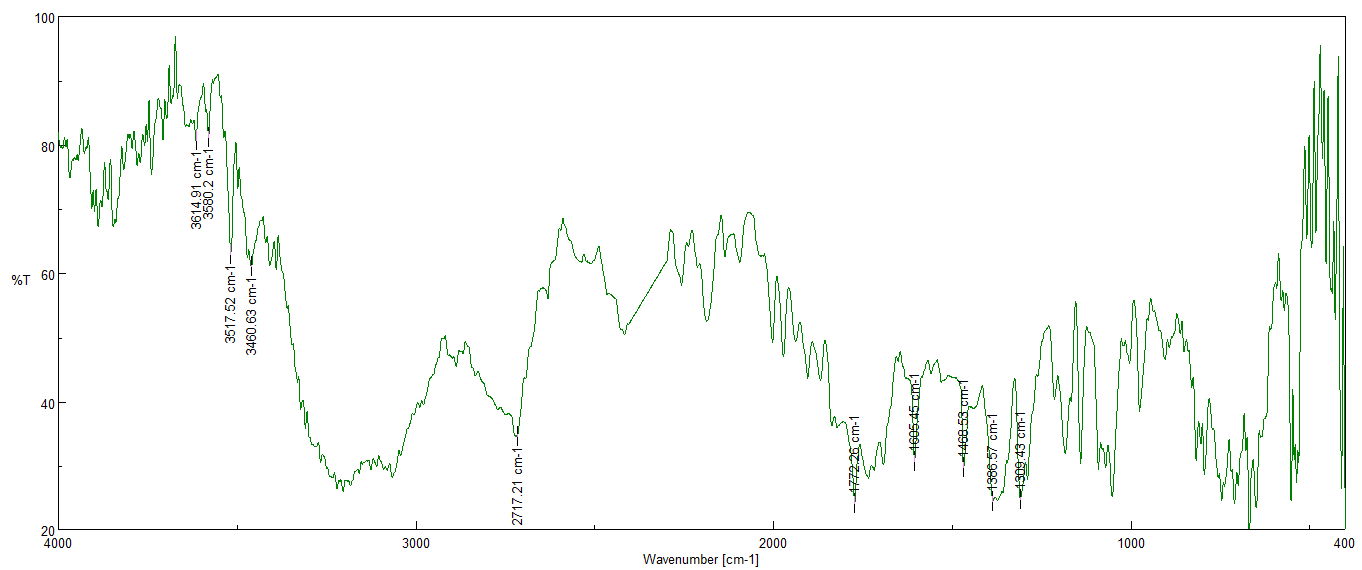
**

**NMR spectra of compound 3:**

**
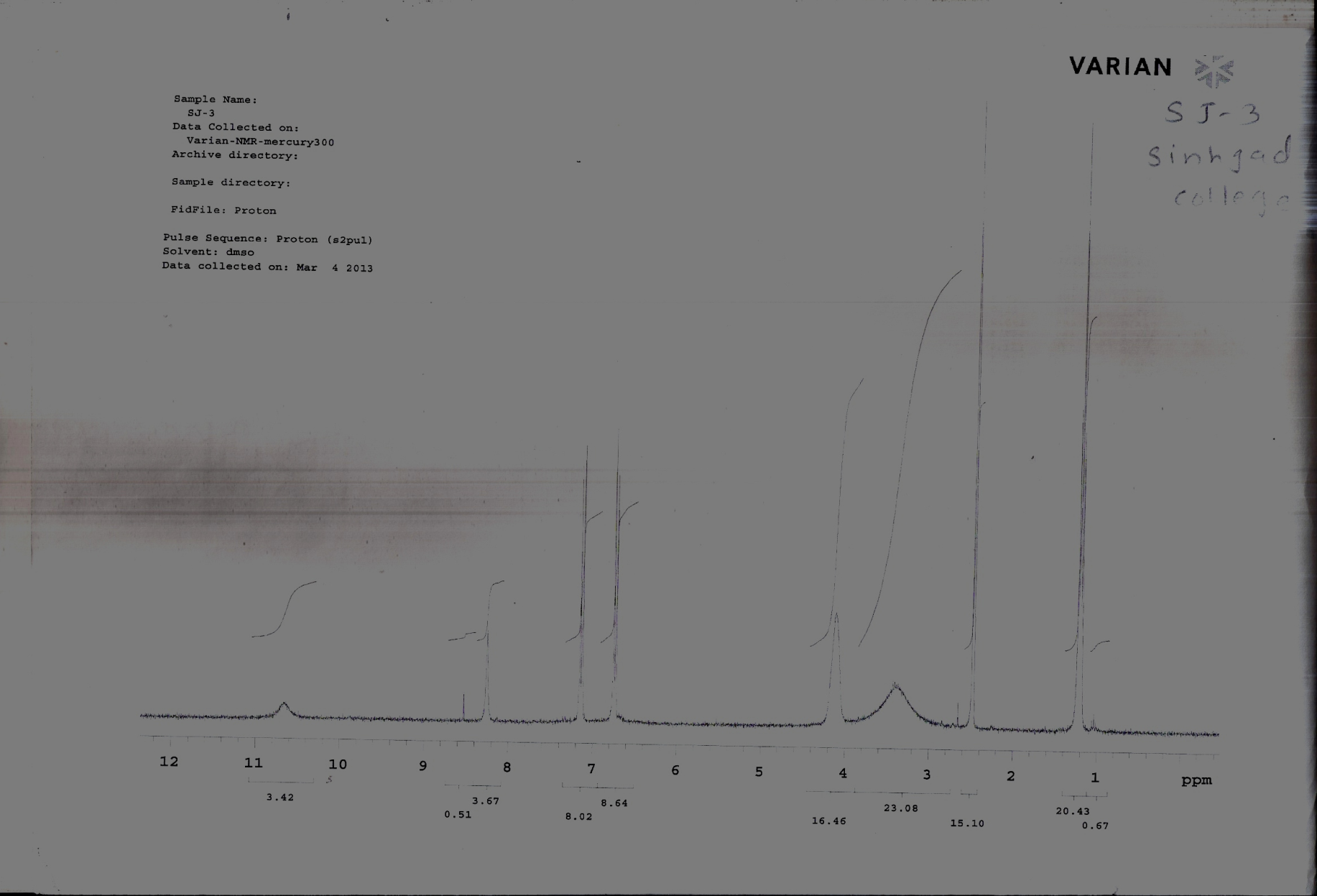
**

**IR spectra of compound 4:**

**
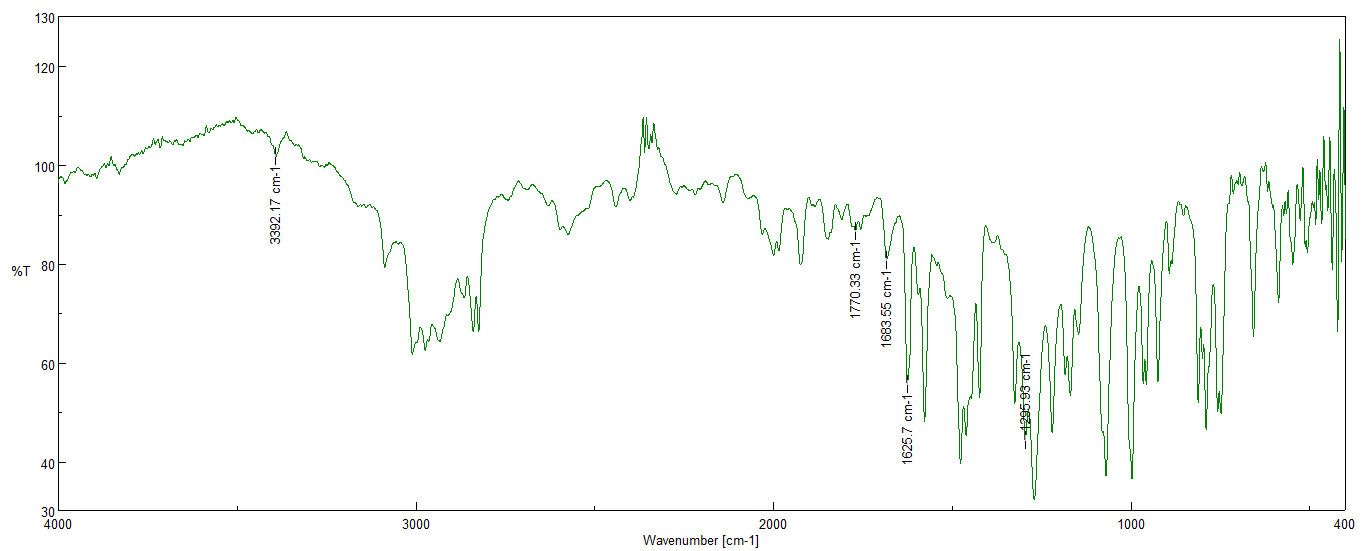
**

**FTIR studies for compound 5:**

**
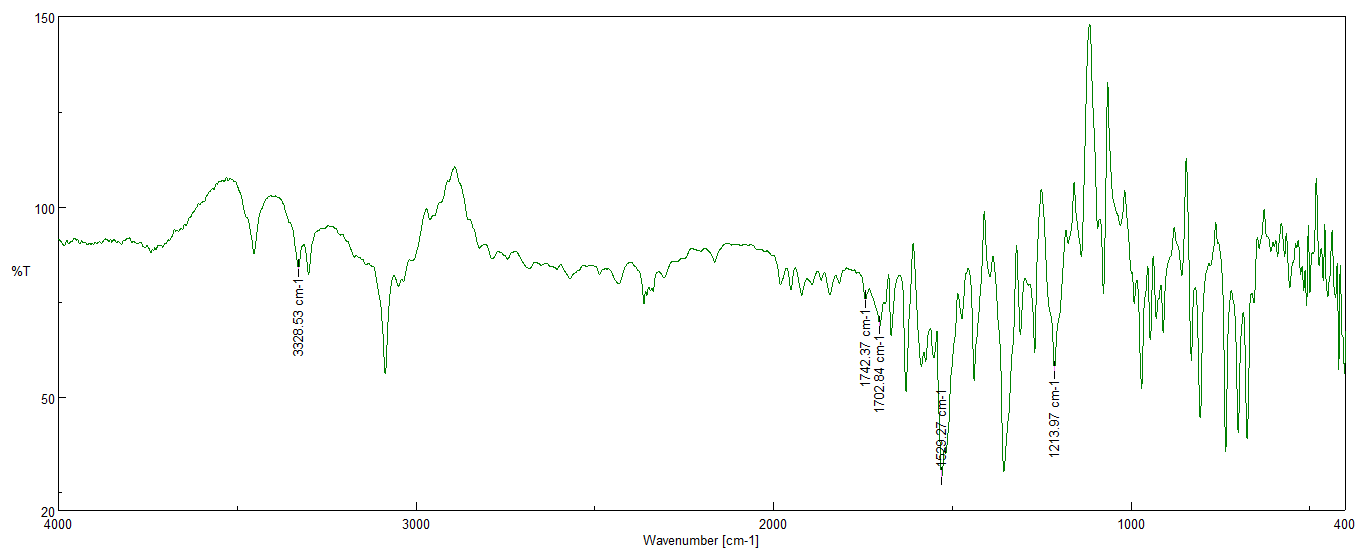
**

**NMR spectra of compound 5:**


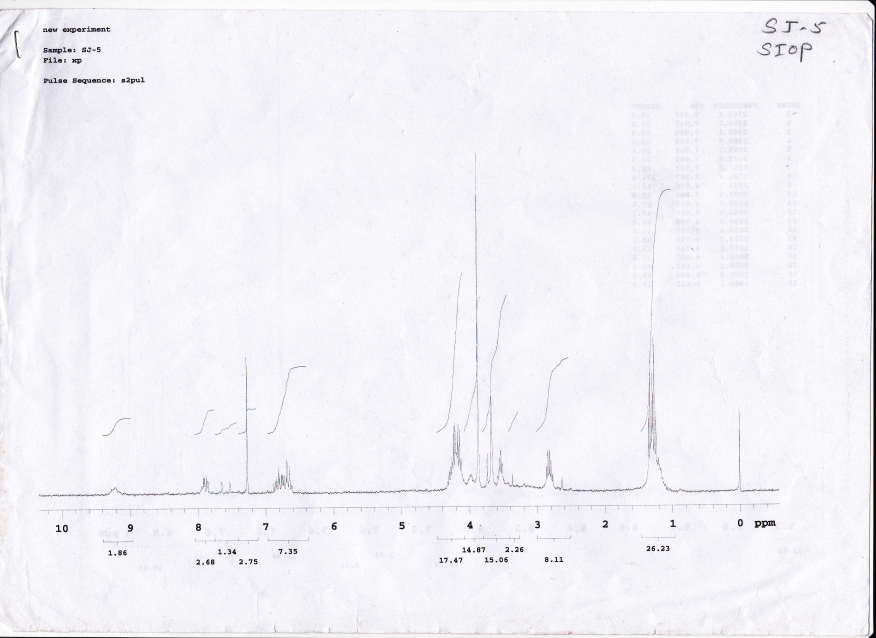


**FTIR studies of SJ-6**

**
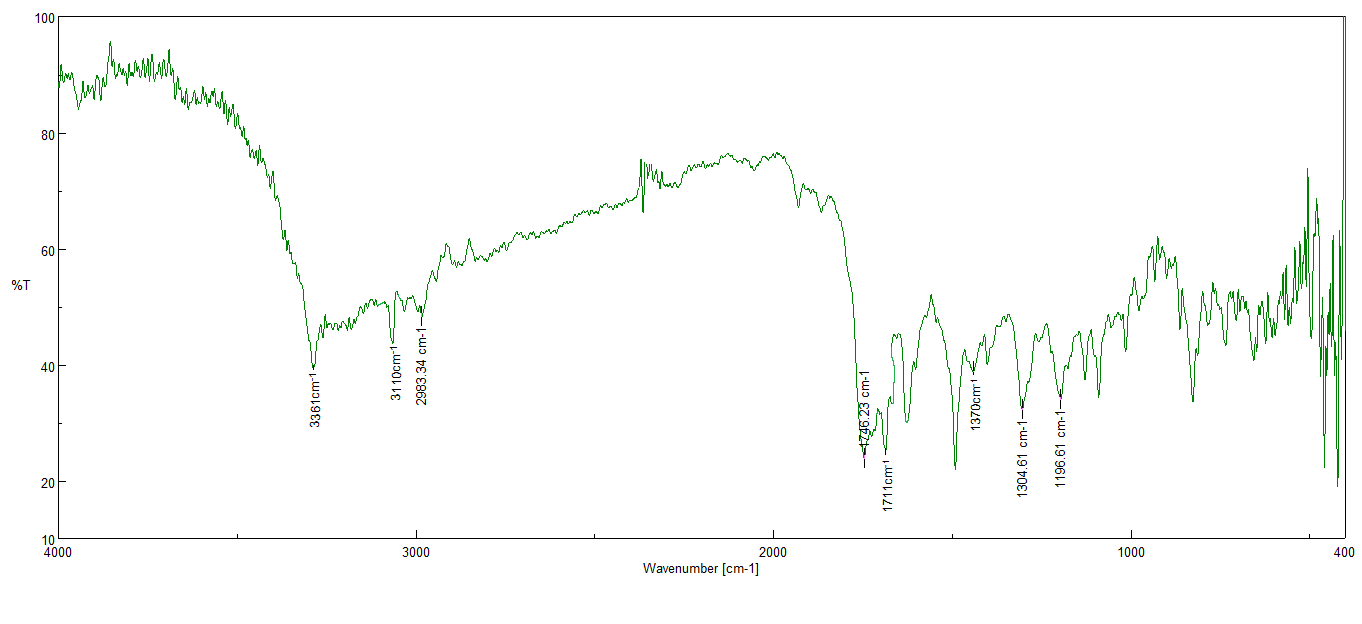
**
